# mTORC1 Dependent Regulation of REDD1 Protein Stability

**DOI:** 10.1371/journal.pone.0063970

**Published:** 2013-05-22

**Authors:** Chia Yee Tan, Thilo Hagen

**Affiliations:** Department of Biochemistry, Yong Loo Lin School of Medicine, National University of Singapore, Singapore, Singapore; German Cancer Research Center, Germany

## Abstract

REDD1 is known to be transcriptionally upregulated in hypoxia. During hypoxic stress, REDD1 plays an important role as a mediator of mTORC1 inhibition. REDD1 is also subject to highly dynamic transcriptional regulation in response to a variety of other stress signals. In addition, the REDD1 protein is highly unstable. However, it is currently not well understood how REDD1 protein stability is regulated. In this study, we discovered that mTORC1 regulates REDD1 protein stability in a 26S proteasome dependent manner. Inhibition of mTORC1 resulted in reduced REDD1 protein stability and a consequent decrease in REDD1 expression. Conversely, activation of the mTORC1 pathway increases REDD1 protein levels. We show that REDD1 degradation is not regulated by HUWE1, Cul4a or other Cullin E3 ubiquitin ligases. Our study shows that mTORC1 increases REDD1 protein stability and reveals a novel mTORC1-REDD1 feedback loop. This feedback mechanism may limit the inhibitory action of REDD1 on mTORC1.

## Introduction

The mechanistic target of rapamycin complex I (mTORC1) is a key regulator of cell proliferation. Its main function is to regulate protein synthesis through phosphorylation of its downstream targets S6 kinase 1 (S6K1), eukaryotic initiation factor 4E (eIF4E)-binding protein 4E-BP1 and eukaryotic elongation factor 2 kinase (eEF2K) [1], [2]. Under conditions of stress unfavorable for cell growth, the mTORC1 pathway is inhibited, leading to reduced rates of protein synthesis and hence conservation of energy resources.

REDD1 (Regulated in Development and DNA Damage responses 1) is a negative regulator of mTORC1 in hypoxia and functions in a TSC2 dependent manner [3], [4]. REDD1 was first identified to be upregulated in response to hypoxia and DNA damage [5], [6]. The *REDD1* gene (also known as RTP801/Dig1/DDIT1) belongs to a family of genes that includes its paralog *REDD2* (TRP801L/Dig2/DDIT4L) [6] and the Drosophila orthologs *Scylla* and *Charybdis* [4]. REDD1 is ubiquitously expressed and is found in most adult tissues, however the expression of REDD2 highly restricted [4]–[6]. *REDD1* is upregulated through transcriptional mechanisms in response to different stress stimuli such as DNA damage, ER stress, hypoxia, serum deprivation, glucocorticoid-, hydrogen peroxide-, dexamethasone-treatment [5]–[9]. Moreover, the induction of *REDD1* is mediated by different transcription factors including p53, p63, ATF4, Sp1 and HIF1, indicating that REDD1 is an important regulator in response to diverse stress conditions [5]–[8].

REDD1 is a highly unstable protein. The REDD1 protein half-life has been reported to be between 5–7 mins [10], [11]. This indicates that REDD1 is also subject to stringent post-translational control. However, as there are no known structural domains or functional motifs present in REDD1, not much is known about the regulation of REDD1. It has been reported that REDD1 is degraded by the Cul4a (Cullin 4a)-DDB1 (DNA damage-binding protein 1)-ROC1 (regulator of cullins 1)-β-TRCP ubiquitin E3 ligase complex through a phosphorylation dependent mechanism mediated by glycogen synthase kinase 3β (GSK3β) [11]. However, it is not known how the stability of REDD1 is regulated in response to physiological signals.

In this study, we identified a novel mTORC1-REDD1 feedback loop whereby mTORC1 regulates REDD1 protein stability. Furthermore, we observed that REDD1 stability is not regulated by GSK3β dependent phosphorylation and that REDD1 is not ubiquitinated by Cul4a or other Cullin RING E3 ubiquitin ligases.

## Results

### mTORC1 Regulates Cellular REDD1 Protein Levels

**Figure 1:**
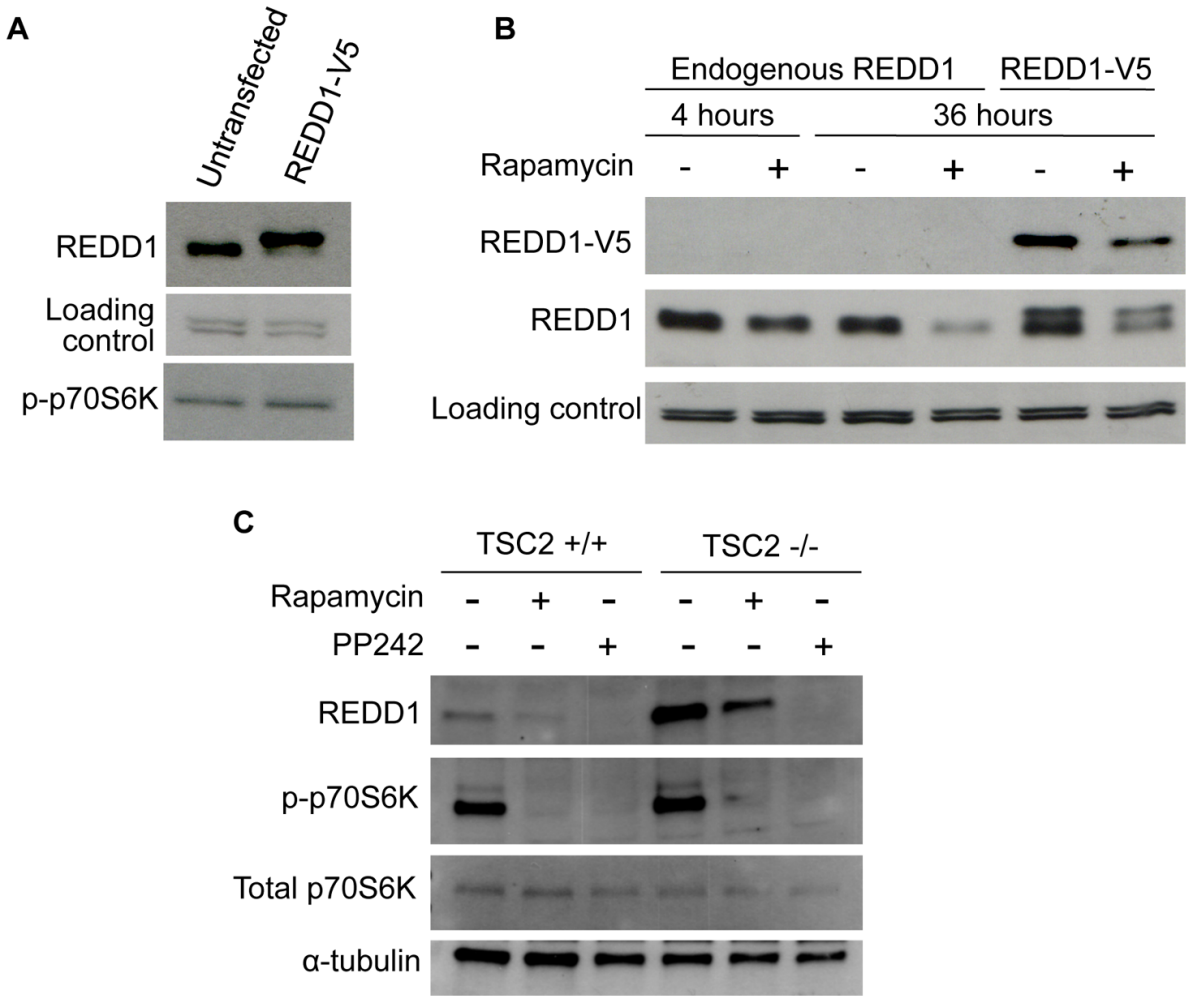
Negative feedback regulation of REDD1. (**A**) REDD1-V5 pcDNA3 (0.4 µg) was transfected in HEK293 cells for 3 days followed by cell lysis and detection of endogenous REDD1 proteins by Western blotting. (**B**) HEK293 cells with and without the transfection of REDD1-V5 pcDNA3 (0.15 µg) were treated with 20 nM rapamycin for 4 or 36 hours followed by cell lysis. (**C**) MEF TSC2^+/+^ and TSC2^−/−^ cells were treated with rapamycin (40 nM) or PP242 (2 µM) for 24 hours before cell lysis. Detection of V5 tag, REDD1, α-tubulin, phosphorylated and total S6 Kinase 1 was performed by Western blotting as described in Materials and methods.

We initially observed that overexpression of REDD1-V5 in HEK293 cells led to reduced levels of endogenous REDD1 protein when compared to REDD1 protein in untransfected cells (Figure 1A). High levels of REDD1 are known to inhibit the mTORC1 pathway. This suggested that inhibition of mTORC1 activity may be responsible for the downregulation of the REDD1 protein levels. We therefore tested if inhibition of the mTORC1 pathway with rapamycin causes a similar downregulation of the REDD1 protein. Indeed, treatment of cells with rapamycin resulted in a marked reduction in REDD1 protein abundance (Figure 1B). To test if the mTORC1 regulation of REDD1 is due to a decrease in transcription, we also determined the effect of mTORC1 inhibition on transfected REDD1 levels as the expression of REDD1-V5 pcDNA3 plasmid is driven by a constitutively active CMV promoter. Transfected REDD1 levels also decreased with rapamycin treatment (Figure 1B). This indicates that the mTORC1 regulation of REDD1 is independent of transcription and is likely a result of altered REDD1 degradation.

To further confirm the effect of mTORC1 activity on REDD1 protein concentrations, we used TSC2^+/+^ and TSC2^−/−^ mouse embryonic fibroblasts (MEFs). These cells have differential mTORC1 activities due to the presence or absence of the mTORC1 upstream negative regulator TSC2. In the TSC2^−/−^ MEFs where mTORC1 is constitutively active, REDD1 levels are much higher compared to TSC2^+/+^ cells (Figure 1C). Thus, consistent with our hypothesis, increased mTORC1 activity led to higher REDD1 protein abundance. As expected from the results in HEK293 cells, treatment with the mTORC1 inhibitors rapamycin or PP242 reduced REDD1 expression markedly in both TSC2^+/+^ and TSC2^−/−^ MEFs (Figure 1C). Taken together, these results indicate that REDD1 protein levels are regulated by mTORC1 activity.

### mTORC1 Regulates REDD1 Protein Stability

**Figure 2:**
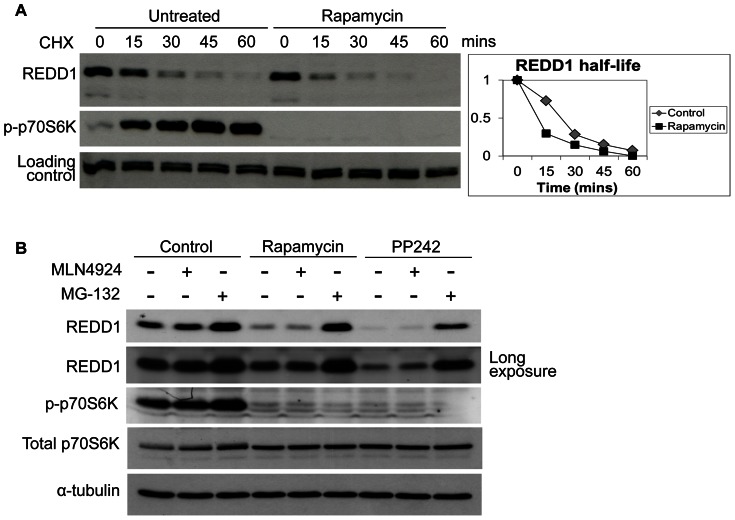
mTORC1 inhibition increases REDD1 degradation. (**A**) HEK293 cells were pretreated with 20 nM rapamycin for 1 hour followed by treatment with cycloheximide (40 µM) and cell lysis at the indicated time points. (**B**) Co-treatment of rapamycin (20 nM) or PP242 (2 µM) with MLN4924 (2 µM) or MG-132 (20 µM) were performed in HEK293 cells as indicated for 4 hours followed by cell lysis.

We next determined whether the downregulation of REDD1 upon mTORC1 inhibition is due to increased protein turnover. To this end, we treated cells with the protein synthesis inhibitor cycloheximide in the presence or absence of rapamycin and determined protein degradation rates. REDD1 half-life was shorter when mTORC1 was inhibited with rapamycin (Figure 2A). In control cells, REDD1 protein decreased by 27% after 15 mins of cycloheximide treatment. In contrast, in the presence of rapamycin, the decrease in REDD1 protein during the same time period was much more dramatic (70%). Furthermore, the decrease in REDD1 levels upon treatment with mTORC1 inhibitors rapamycin and PP242 treatment was reversed with addition of proteasome inhibitor, MG-132 (Figure 2B). This indicates that upon mTORC1 inhibition REDD1 is degraded in a 26S proteasome dependent manner.

### Regulation of REDD1 by the HUWE1 E3 Ubiquitin Ligase

**Figure 3:**
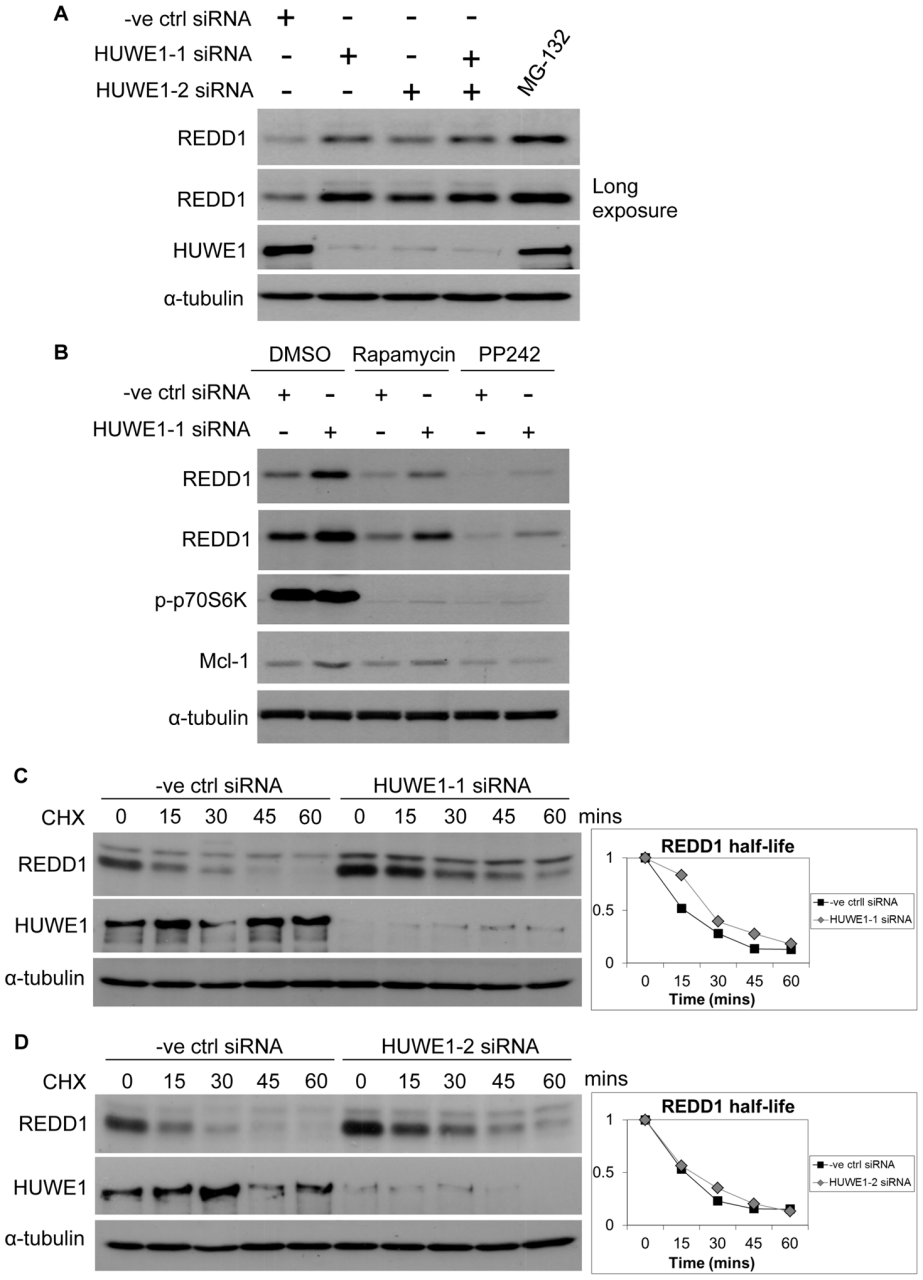
REDD1 stability is regulated by HUWE1 ubiquitin ligase. (**A**) HEK293 cells were transfected with 20 nM negative control siRNA or HUWE1−1 or −2 siRNAs in the indicated combinations for 3 days and MG-132 (20 µM) was added to cells for 4 hours followed by cell lysis. (**B**) HEK293 cells were transfected with 20 nM control or HUWE1 siRNAs for 3 days and rapamycin (40 nM) or PP242 (2 µM) were added to cells for 8 hours followed by cell lysis. (**C**,**D**) HEK293 cells were transfected with 20 nM negative control or HUWE1-1 (**C**) or −2 (**D**) siRNAs for 3 days followed by cycloheximide (40 µM) treatment and cell lysis at indicated time points.

To identify the E3 ligase involved in the REDD1 degradation upon mTORC1 inhibition, we tested a number of candidates. Interestingly, we observed that the knockdown of the E3 ubiquitin ligase HUWE1 caused an increase in REDD1 steady state levels (Figure 3A and B). This effect was not due to changes in mTORC1 activity, as indicated by the unaltered levels of p70 S6 kinase phosphorylation (Figure 3B). An increase in the steady state level of the known HUWE1 substrate Mcl-1 served as a positive control (Figure 3B). We also observed that when mTORC1 activity was inhibited with rapamycin or PP242, REDD1 protein levels were higher in cells transfected with HUWE1 siRNA compared to control cells (Figure 3B). However, the increase in REDD1 protein levels upon HUWE1 knockdown was similar in untreated cells (Figure 3B). Based on these results, we concluded that HUWE1 is not involved in the degradation of REDD1 upon inhibition of mTORC1.

Given that HUWE1 knockdown caused an increase in the steady state levels of the REDD1 protein, we next tested whether the HUWE1 is involved in the basal turnover of REDD1. We measured the protein half-life using cycloheximide chase with two different HUWE1 siRNAs. However, in both experiments there was no significant change in the half-life of REDD1 in cells with HUWE1 knockdown compared to controls (Figures 3 C and D). This suggests that HUWE1 regulates REDD1 via a mechanism that is independent of protein degradation.

### REDD1 Protein Stability is not Regulated by Cullin E3 Ubiquitin Ligases

Cullin E3 ubiquitin ligases constitute approximately half of the cellular E3 ligases. Furthermore, Cullin 4 E3 ligase has previously been implicated in regulating the basal turnover of REDD1. We therefore determined whether the family of Cullin E3 ligases is involved in REDD1 degradation upon mTORC1 inhibition. This was tested using MLN4924, an inhibitor of Nedd8 E1 activating enzyme which inhibits all cullin E3 ligases [12]. We observed that MLN4924 did not reverse the degradation of REDD1 upon treatment of cells with the mTORC1 inhibitors rapamycin or PP242 (Figure 2B). Furthermore, unlike the proteasome inhibitor MG-132, MLN4924 also had no effect on the basal protein levels of REDD1 (Figure 2B). This result was surprising given the previous report of Cullin 4 dependent regulation of REDD1 protein stability [11]. Hence, in further studies, we characterized the involvement of Cullin E3 ligases in the regulation of REDD1 stability in detail.

**Figure 4:**
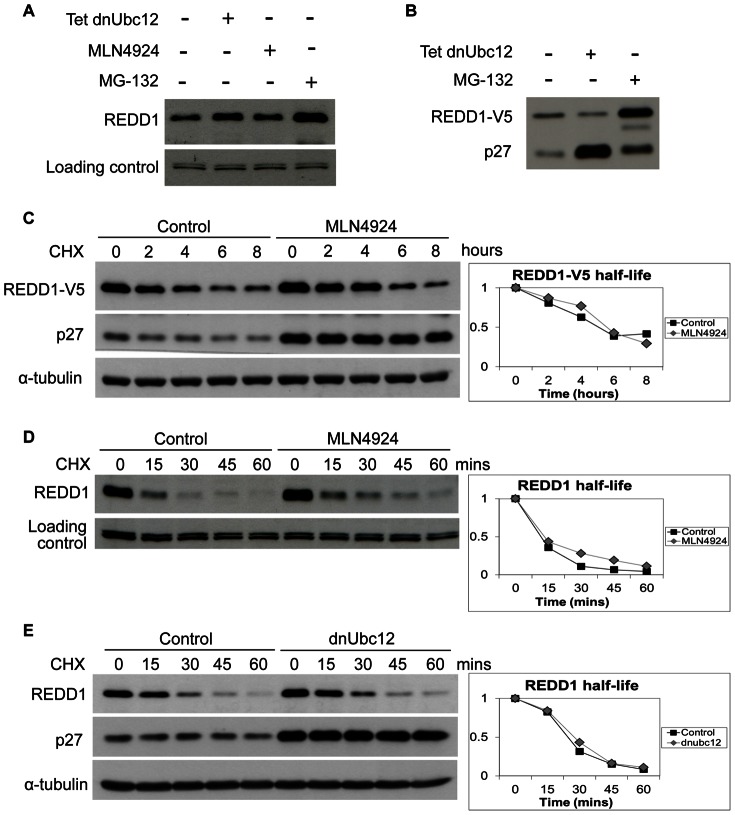
REDD1 is not regulated by Cullin E3 Ubiquitin ligases. (**A**,**B**) Untransfected (**A**) or REDD1-V5 pcDNA3 (0.3 µg) transfected (**B**) HEK293 cells stably expressing tetracycline inducible dnUbc12-HA were induced with 1 µg/ml tetracycline for 24 hours (**A**,**B**) or treated with 3 µM MLN4924 (**A**) or 20 µM MG-132 (**A**,**B**) for 8 hours followed by cell lysis. (**C**,**D**) Untransfected HEK293 (**C**) or HEK293 transfected with REDD1-V5 pcDNA3 (0.3 µg) (**D**) were pre-treated with 3 µM MLN4924 followed by cycloheximide (40 µM) treatment and cell lysis at the indicated time points. (**E**) HEK293 cells stably expressing tetracycline inducible dnUbc12-HA were induced with 1 µg/ml tetracycline for 24 hours followed by cycloheximide (40 µM) treatment and cell lysis at the indicated time points.

As an alternative approach to using MLN4924, we utilized a genetic approach by expressing the dominant negative C111S mutant Ubc12 (dnUbc12) conjugating enzyme, induced with tetracycline, in HEK293 cells [13]. The dnUbc12 cells have a defective neddylation pathway and this leads to the inactivation of the Cullin RING E3 ubiquitin ligases. Similar to MLN4924 treatment, induction of dnUbc12 did not have any significant effect on both endogenous and transfected REDD1 proteins (Figures 4A and B). Overexpression of dnUbc12 caused a dramatic increase in the known Cullin E3 ligase substrate p27 which was even more pronounced compared to the proteasome inhibitor MG-132 (Figure 4B). This shows that REDD1 steady state level is not regulated by Cullin ubiquitin ligases. We subsequently performed experiments to determine the effect of pharmacological and genetic inhibition of Cullin E3 ligases on the REDD1 protein half-life. We observed no difference in the half-life of both endogenous and transfected REDD1 when HEK293 cells were treated with MLN4924 (Figures 4C and D) or when dnUbc12 expression was induced with tetracycline (Figure 4E). In contrast, as expected, p27 was stabilized with both inhibitors. These results indicate that REDD1 stability is not regulated by Cullin RING E3 ubiquitin ligases.

### Both Cul4a and Phosphorylation of REDD1 by GSK3β are not Involved in Basal REDD1 Protein Turnover

**Figure 5:**
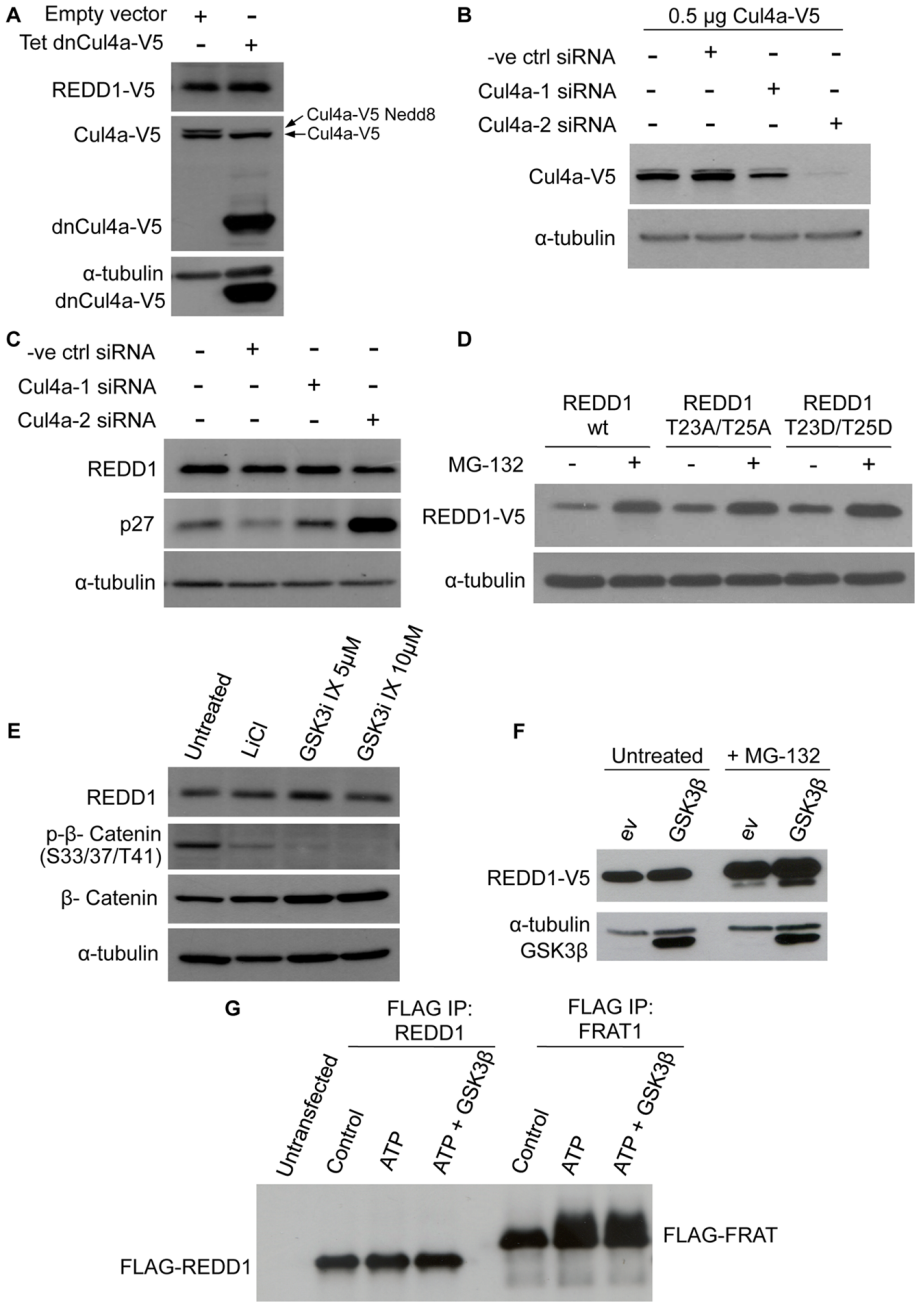
REDD1 is not degraded by Cul4a or phosphorylated by GSK3β at Thr 23 and Thr25. (**A**) HEK293 cells were transfected with empty vector or tetracycline inducible dnCul4a-V5 pcDNA4/TO (1 µg) and Cul4A-V5 pcDNA3 (0.03 µg) followed by tetracycline (1 µg/ml) induction for 24 hours and cell lysis. (**B**) HEK293 cells were transfected with 0.5 µg Cul4a-V5 pcDNA3 for 15 hours followed by transfection of 20 nM control or Cul4a siRNAs to determine siRNAs efficiency. (**C**) HEK293 cells were transfected with 20 nM control or Cul4a siRNAs for 3 days followed by cell lysis. (**D**) REDD1-V5 pcDNA3 wild type, T23A T25A or T23D T25D plasmids (0.4 µg) were transfected in HEK293 cells for 3 days and treated with 20 µM MG-132 for 6 hours followed by cell lysis. (**E**) HEK293 cells were treated with 30 mM LiCl or GSK3 inhibitor IX (5 µM or 10 µM) for 20 hours followed by cell lysis. (**F**) HEK293 cells were co-transfected with 0.2 µg REDD1-V5 pcDNA3 and 0.3 µg GSK3β pcDNA3 or empty pcDNA3 for 3 days followed by MG-132 (20 µM) treatment for 6 hours followed by cell lysis. (**G**) HEK293 cells were transfected with 3 µg FLAG-REDD1 or FLAG-FRAT1 for 3 days followed by cell lysis and FLAG immunoprecipitation. In vitro phosphorylation of REDD1 and FRAT1 was carried out as described in Materials and Methods.

It has been reported that REDD1 degradation is mediated by the Cul4a E3 ubiquitin ligase complex and that this in turn is dependent on REDD1 phosphorylation at Thr23 and Thr25 sites by GSK3β. Therefore, to confirm this, we overexpressed a tetracycline inducible dominant negative Cul4a-V5 plasmid (dnCul4a, amino acids 1–439) in HEK293 cells [14]. dnCul4a is able to interact with substrate proteins, but unable to recruit the ubiquitin-charged E2 ubiquitin conjugating enzyme. As a result, dnCul4a sequesters substrates in inactive complexes and inhibits their ubiquitination and degradation. If REDD1 is a substrate of Cul4A, we would expect to see an increase in REDD1 expression with induction of dnCul4A. However, it was observed that REDD1 levels were not affected by dnCul4a induction (Figure 5A). To confirm that dnCul4a overexpression and induction was able to inhibit Cul4a function, we demonstrated that dnCul4a markedly reduced the neddylation levels of coexpressed full length Cul4a (Figure 5A). Nedd8 modification (neddylation) of cullin proteins is required for the function of all cullin E3 ligases. Cullin neddylation is dependent on the binding of substrate proteins [14]. Hence, the markedly reduced neddylation of full length Cul4a indicates that substrate binding is inhibited. The dnCul4a experiment suggests that REDD1 protein stability is not regulated by Cul4a.

We also used siRNA mediated silencing of Cul4a to confirm the results obtained with the dnCul4a cell line. We used two different siRNA oligonucleotides and Cul4a siRNA-2 proved more effective (Figure 5B). We observed that knockdown of Cul4a did not affect REDD1 expression (Figure 5C). As expected, the level of the Cul4a-DDB1-DDB2 substrate p27 level was markedly increased with Cul4a siRNA-2. In conclusion, Cul4a is unlikely to be involved in the regulation of REDD1 protein stability.

It has been reported that GSK3β phosphorylates REDD1 at residues Thr23 and Thr25, resulting in REDD1 recruitment to the Cullin 4a-β-Trcp E3 ligase complex. To confirm involvement of these threonine residues in the regulation of REDD1 protein stability, we mutated both Thr23 and Thr25 to alanines. These mutations would be expected to stabilize REDD1 protein. However, we did not observe any significant difference in the stability of REDD1 mutant compared to controls (Figure 5D). Addition of proteasome inhibitor caused a similar increase in wild type and T23A/T25A mutant REDD1 protein levels, suggesting that the phosphorylation sites are not important for the regulation of REDD1 protein stability. Furthermore, mutation of the threonine 23 and 25 residues to aspartate to mimic phosphorylation also did not have any effect on REDD1 stability (Figure 5D). To further confirm that GSK3β does not regulate REDD1 protein levels, two different inhibitors of GSK3β were added to HEK293 cells, lithium chloride and GSK3 inhibitor IX. Both inhibitors blocked the activity of GSK3β, as indicated by the marked decrease in the phosphorylation of the GSK3β substrate β-catenin (Figure 5E). Inhibition of GSK3β is expected to increase REDD1 stability. However, we did not observe any significant difference in REDD1 expression levels upon addition of the GSK3β inhibitors (Figure 5E). Moreover, overexpression of GSK3β did not result in decreased REDD1 stability despite a dramatic increase in GSK3β expression compared to untransfected cells (Figure 5F). We also tested whether incubation of REDD1 with recombinant GSK3 causes a band shift indicative of phosphorylation. Of note, Katiyar *et al*. reported that GSK3β dependent phosphorylation of REDD1 causes a faster migration in SDS gels [11]. However, no change in electrophoretic mobility was detected, whereas the reported GSK3β substrate FRAT1 displayed the expected band shift (Figure 5G). Taken together, our results suggest that both basal and mTORC1 regulated REDD1 degradation is mediated via a novel mechanism that does not involve Cullin E3 ligases and GSK3β dependent phosphorylation.

## Discussion

**Figure 6:**
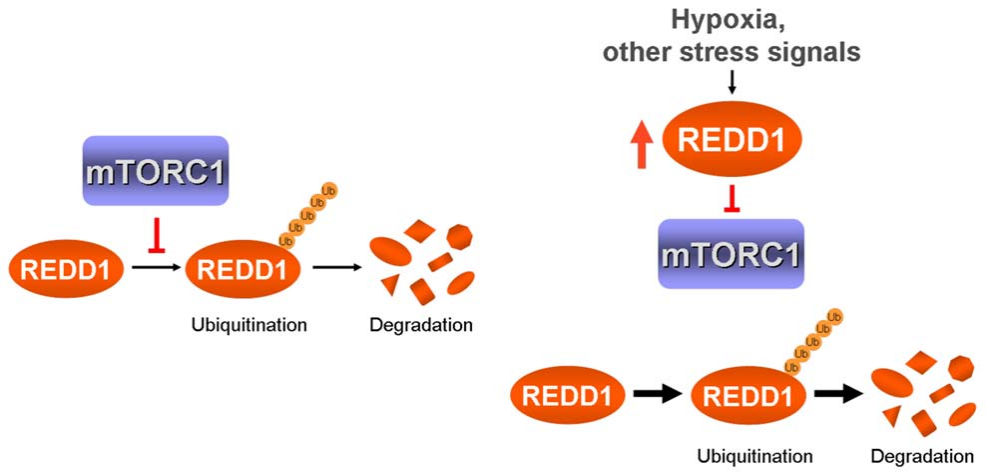
The mTORC1-REDD1 limits the inhibitory action of REDD1 on mTORC1. When mTORC1 is active, ubiquitination and proteasome dependent degradation of REDD1 is inhibited (left panel). Under conditions of hypoxia and other stress stimuli, REDD1 is transcriptionally induced, leading to mTORC1 inhibition. As a consequence, REDD1 ubiquitination and degradation is no longer restricted by mTORC1 (right panel). This mTORC1-REDD1 feedback mechanism limits the inhibitory action of REDD1.

REDD1 is an important negative regulator of mTORC1 in response to stress. In this study, we have shown that mTORC1 in turn also regulates REDD1. mTORC1 dependent regulation of REDD1 is at the level of the REDD1 protein stability. Inhibition of mTORC1 using the small molecules rapamycin and PP242 or by overexpressing REDD1 led to reduced REDD1 protein stability and a consequent decrease in REDD1 expression. This mTORC1-REDD1 feedback loop would limit the inhibitory action of REDD1 on mTORC1 (Figure 6). The physiological significance of the mTORC1-REDD1 feedback mechanism is currently not clear and requires further study. However, our finding highlights that in addition to the extensive transcriptional control of REDD1, the REDD1 protein is also subject to posttranslational regulatory mechanisms.

In further experiments, we studied the mechanism through which REDD1 stability is regulated. We found that REDD1 degradation in response to mTORC1 inhibition is proteasome dependent. However, contrary to a previous report [11], we observed that mTORC1 protein stability is not controlled by the Cul4a E3 ubiquitin ligase. The discrepancy between the reported Cul4a dependent regulation of REDD1 and our results is unlikely to be due to differences in cell type as both studies used HEK293 cells. Furthermore, we were unable to confirm the role of GSK3β in targeting REDD1 for ubiquitination. Thus, we found that mutation of the reported GSK3β phosphorylation sites in REDD1 to alanine or aspartate did not affect REDD1 protein levels. Similarly, inhibition of GSK3β with two different inhibitors and overexpression of GSK3β were without effect on REDD1 protein expression. Furthermore, no band shift was observed upon incubation of REDD1 with GSK3β in vitro. It should be noted that mutation of serine or threonine residues to aspartate does not always have phospho-mimetic effects and phosphorylation events do not cause a lower mobility in every instance. Despite these limitations, when taking all of our findings together, there is strong evidence against a role of GSK3β in the regulation of REDD1 protein stability.

Our results suggest that an alternative E3 ligase is responsible for both basal REDD1 ubiquitination and ubiquitination that is induced upon mTORC1 inhibition. Using pharmacological and genetic inhibitory approaches, we have ruled out any role for members of the Cullin RING E3 ligase family, which constitutes about half of all cellular E3 ubiquitin ligases. Although knockdown of the HUWE1 E3 ubiquitin ligase resulted in increased REDD1 protein steady state levels, our further studies indicated that this effect is not due to an effect of HUWE1 on REDD1 protein stability. Hence, the identity of the E3 ligase that mediates basal REDD1 ubiquitination and ubiquitination upon mTORC1 inhibition is currently unknown. Identification of this E3 ligase is important as this ligase may be a unique drug target for specific inhibition of mTORC1.

## Materials and Methods

### Cell Culture and Transfection

TSC2^+/+^-*p53* ^−/−^ and TSC2^−/−^ *p53* ^−/−^ MEFs were kindly provided by D.J. Kwiatkowski (Brigham and Women’s Hospital, Harvard Medical School, Boston, MA) [15]. The Human Embryonic Kidney (HEK293) (ATCC and Invitrogen), TSC2^+/+^-*p53* ^−/−^ and TSC2^−/−^ *p53* ^−/−^ MEF cells were cultured in Dulbecco’s modified Eagle’s medium (DMEM) supplemented with 10% inactivated fetal bovine serum, 2 mM L-glutamine and 1% penicillin-streptomycin (Invitrogen) and maintained in a 5% CO_2_ incubator at 37°C. For overexpression experiments, sub-confluent cells were transfected using Genejuice (Novagen) according to the manufacturer's instructions. Knockdown experiments using siRNAs (predesigned dsiRNAs, IDT) were performed using Lipofectamine RNAiMax (Invitrogen) according to the instructions by the manufacturer.

### Plasmid Constructs

The human REDD1-V5 pcDNA3 plasmid was constructed by PCR amplification from human brain cDNA and subsequently inserted into the pcDNA3 vector with a C terminal V5 tag using KpnI and XbaI restriction sites with a SacII restriction site inserted between REDD1 and the V5 tag. Mutagenesis of REDD1 Threonines 23 and 25 to Alanines was carried out using the Stratagene site-directed mutagenesis kit. The HA-p70S6K1 T389D pcDNA3 and HA-p70S6K1 T389A pcDNA3 plasmids were constructed from the pRK7-HA-S6K1-WT (Addgene Plasmid 8984) [16]. The HA-p70S6K1 gene was digested from the pRK7-HA-S6K1-WT plasmid using XbaI and EcoRI restriction enzymes and ligated in to the pcDNA3.1 (-). Mutagenesis of p70S6K1 Threonine 289 site to Alanine or aspartate was carried out using the Stratagene site-directed mutagenesis kit. GSK3β plasmid was previously described [17]. The tetracycline-inducible dnUbc12 (C111S) and dnCul4a (amino acids 1 to 439) cell lines were generated using the T-Rex system (Invitrogen) according to the manufacturer’s instructions, as previously described [13], [14].

### *In vitro* Phosphorylation of REDD1 and FRAT1

FLAG-immunoprecipitates (REDD1-FLAG or FRAT-FLAG) from HEK293 cell lysates were incubated on a shaking platform for 45 minutes at room temperature in 50 mM Tris pH 7.5, 25 mM MgCl_2_ and 2 mM DTT in the presence or absence of 1 mM ATP and/or the recombinant protein GSK3β. Following the reaction, the samples were denatured in SDS-sample buffer and subjected to SDS-PAGE and immunoblotting.

### Immunoblotting

Whole cell lysates were prepared by rinsing the cells in ice cold 1x PBS followed by cell lysis using lysis buffer with the following composition: 25 mM Tris-HCl (pH 7.5), 100 mM NaCl, 2.5 mM EDTA, 2.5 mM EGTA, 20 mM NaF, 1 mM Na_3_VO_4_, 20 mM sodium β-glycerophosphate, 10 mM sodium pyrophosphate, and 0.5% Triton X-100 containing freshly added protease inhibitor cocktail (Roche Diagnostics) and 0.1% β-mercaptoethanol. Equal amounts of protein from each sample were separated by SDS–PAGE (10%) and transferred onto nitrocellulose membranes. The blots were probed with a primary antibody followed by a secondary antibody conjugated to horseradish peroxidase. The following primary antibodies were used: rabbit anti-REDD1 (10638-1-AP; Proteintech), rabbit anti-phospho-p70 S6 kinase (Thr389) (9234; Cell Signaling), rabbit anti-p70 S6 kinase (9202; Cell Signaling), mouse anti-HIF-1α (610959; BD Biosciences), mouse anti-p27 (610241; BD Biosciences), mouse anti-HECTH9 (AX8D1)/HUWE1 (5695; Cell Signaling), mouse anti-GSK3β (610202; BD Biosciences), mouse anti-α-tubulin (236–10501; Molecular Probes, Invitrogen), mouse anti-V5 (MCA1360; AbD Serotec), mouse anti-Mcl-1 (sc-12756; Santa Cruz Biotechnology). Protein levels on the blots were detected using the enhanced chemiluminescence system (GE Healthcare) according to the manufacturer’s instructions. All western blots are representative of at least two independent experiments.
